# Neutrophil Extracellular Trap Formation in Advanced Heart Failure Patients—Preliminary Report

**DOI:** 10.3390/ijms25179633

**Published:** 2024-09-05

**Authors:** Tomasz Urbanowicz, Anna Olasińska-Wiśniewska, Ewelina Wojtasińska, Krzysztof J. Filipiak, Małgorzata Tomaszewska, Jędrzej Sikora, Marta Krama, Zofia Radek, Kajetan Grodecki, Aleksandra Krasińska-Płachta, Beata Krasińska, Zbigniew Krasiński, Andrzej Tykarski, Marek Jemielity, Joanna Rupa-Matysek

**Affiliations:** 1 Cardiac Surgery and Transplantology Department, Poznan University of Medical Sciences, 61-107 Poznan, Poland; 2 Department of Hematology and Bone Marrow Transplantation, Poznan University of Medical Sciences, 61-107 Poznan, Poland; 3 Department of Hypertensiology, Angiology and Internal Medicine, Poznan University of Medical Sciences, 61-107 Poznan, Poland; 4 Institute of Clinical Science, Maria Sklodowska-Curie Medical Academy, 00-136 Warsaw, Poland; 5 1st Cardiology Department, Poznan University of Medical Sciences, 61-107 Poznan, Poland; 6 1st Cardiology Department, Warsaw University of Medical Sciences, 02-091 Warsaw, Poland; 7 Department of Ophthalmology, Poznan University of Medical Sciences, 61-107 Poznan, Poland; 8 Department of Vascular, Endovascular Surgery, Angiology and Phlebology, Poznan University of Medical Science, 61-848 Poznan, Poland

**Keywords:** neutrophil extracellular trap (NET), citrullinated histone 3 (CH3), heart failure (HF), left ventricular assist device (LVAD), Heartmate 3

## Abstract

In end-stage heart failure, which is characterized by persistent or progressive ventricular dysfunction despite optimal medical therapy, a left ventricular assist device (LVAD) can be beneficial. Congestive heart failure provokes inflammatory and prothrombotic activation. The aim of this study was to evaluate the serum concentration of citrullinated histone 3 (CH3) representing neutrophil extracellular trap (NET) formation in patients referred for LVAD implantation. There were 10 patients with a median age of 61 (57–65) years enrolled in a prospective single-center analysis who underwent LVAD implantation. The CH3 plasma concentration was measured preoperatively and on the 1st and 7th postoperative days, followed by control measurements on the median (Q1–3) 88th (49–143) day. The preoperative CH3 concentration strongly correlated with brain natriuretic peptide (r = 0.879, *p* < 0.001). Significant differences in CH3 serum concentration were observed between pre- and postoperative measurements, including an increase on the first postoperative day (*p* < 0.001), as well as a decrease on the seventh day (*p* = 0.016) and in follow-up (*p* < 0.001). CH3 concentration, as a marker of NET formation, decreases after LVAD implantation.

## 1. Introduction

End-stage heart failure (HF) is characterized by persistent or progressive ventricular dysfunction despite optimal medical therapy [1]. Inotropic therapy and intravenous vasodilators are beneficial in the improvement of hemodynamics, especially in severely decompensated patients [2]. Mechanical support as a bridge to either recovery, heart transplantation, or long-term therapy is indicated to optimize hemodynamics and reverse end-organ damage when medical therapy alone is insufficient [3].

HF, which results in systemic hypoperfusion, provokes inflammatory activation [4], followed by acquired coagulopathy [5].

Neutrophils are first-line responders to injury and infection. Their wide range of effector mechanisms include cytokine release, phagocytosis, reactive oxygen species (ROS) generation, and neutrophil extracellular trap (NET) formation [6]. Their role in chronic heart failure still remains very poorly understood [7]. The web-like structures made of genomic DNA, histones, and granular proteins released by neutrophils as NETs were found to be related to disseminated coagulopathy secondary to inflammatory activation [8].

Increased thromboembolic risk was present in HF patients [9]. Platelet signaling pathway dysregulation related to increased soluble P-selectin, adhesion proteins, and platelet activation markers (CD63, CD40 ligand, and P-selectin) was observed [10].

Unresolved inflammation is a key moderator of congestive heart failure. Molecular patterns related to inflammatory activation are major aspects in initiating and propagating cardiac pathology [11]. In Kumar et al.’s study [12], flow cytometry analysis revealed cytotoxic and inflammatory innate activation in HF patients. The relation between a higher risk of adverse cardiac events in patients with HF and increased neutrophil count combined with their activation was postulated [12]. Neutrophils, upon activation, may create web-like structures named neutrophil extracellular traps (NETs), and the process of their formation is named NETosis [13]. The increased levels of NET components in acute coronary syndrome, which reflected the immune response measured by circulating interleukin (IL) IL-8 levels, were correlated with myocardial function [14]. NETs are created and released via the process of cell death, and there are two distinct types: lytic [15] and excretic [16]. Neutrophil granules such as myeloperoxidase, cathepsin G, and gelatinase, followed by deoxyribonucleic acid (DNA) remnants, were found in NETs [17]. Although standardization problems exist, citrullinated histone 3 (CH3) is one of the most widely used NET markers [18]. In animal studies, the NET formation related to histone citrullination catalyzed by neutrophils’ peptidyl arginine deiminase 4 (PAD4) was presented [19]. This conversion results in weakening histone–DNA binding and the release of decondensed DNA that is a requisite for NET creation [20].

The aim of this study was to present changes in CH3 concentration in patients referred for LVAD implantation due to advanced HF.

## 2. Results

In all patients, Heartmate 3 (Abbott Inc., Chicago, IL, USA) was implanted using a cardiopulmonary bypass application. The postoperative period was uneventful, without postoperative bleeding complications, though temporary renal replacement therapy was necessary in each subject.

The median (Q1–3) postoperative stay was 46 (32–71) days. The pump function was described by a median pump speed setting (Q1–3) of 5150 (3950–5200) revolutions per minute (rpm) and a pulsatility index of 4.25 (4.15–4.30) liters per minute. There were no fatal incidents in the presented group, and the median (Q1–3) follow-up time was 88 (49–143) days. All patients presented functional improvement reflected in New York Heart Association classification (NYHA) change.

### 2.1. Preoperative Patient Analysis

Among laboratory results, the BNP concentration was 1321 (1024–2822) pg/mL, with clinical and laboratory signs of an absence of volume overload, including median hematocrit levels of 36 (34–43)% and sodium concentration of 141 (138–142) mmol/L. Detailed information is presented in Table 1.

In transthoracic echocardiography, patients presented severe left ventricular dysfunction with a median (Q1–3) left ventricular ejection fraction of 20 (17.5–26.0)%.

The right ventricular function was estimated by tricuspid annular plane systolic excursion (TAPSE) with median values of 14 (14–15) mm and a right ventricular diameter of 35 (33–38) mm. The right heart catheterization (RHC) revealed a significantly decreased cardiac index (CI) with median values of 1.8 (1.3–2.0) L/min/m^2^. Patients were characterized by increased pulmonary vascular resistance secondary to left-sided HF with median (Q1–3) values of 293 (224–380) dynes/sec/cm^5^. Patients’ echocardiographic and RHC results are presented in Table 2.

### 2.2. Citrullinated Histone 3 (CH3) Measurements

The citrullinated histone 3 serum concentrations were measured at four time points. Preoperatively, the median CH3 values were 1745 (956–2482) pg/mL. On the first postoperative day, an increase to 3858 (2639–5000) pg/mL was observed. In the postoperative period, a gradual decrease in CH3 concentration was noticed, including the seventh postoperative day (991 (867–2143) pg/mL) and outpatient follow-up (563 (474–615) pg/mL).

### 2.3. Correlation between Preoperative Characteristics and Plasma CH3 Concentration

**Figure 1:**
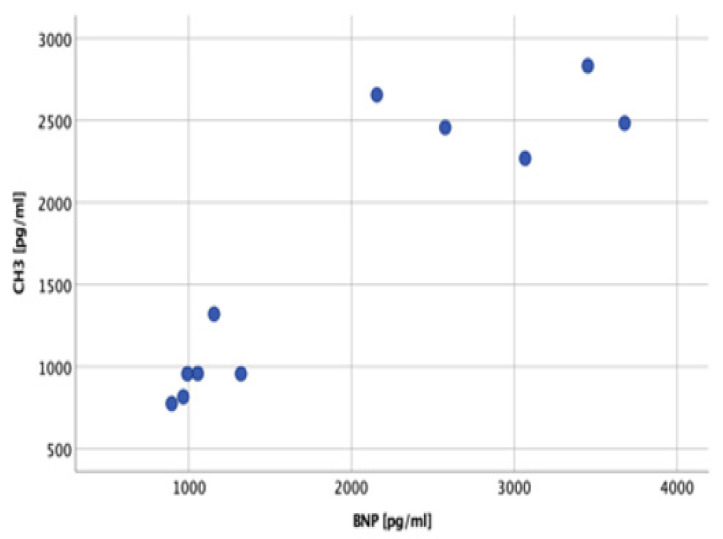
Correlation between CH3 serum concentration and BNP.

The correlations between preoperative laboratory, echocardiographic, and RHC results were analyzed. A strong correlation between the marker of NET formation (CH3 serum concentration) and BNP serum concentration was noticed (r = 0.879, *p* < 0.001), as presented in Figure 1.

### 2.4. Time-Related Changes in Citrullinated Histrone 3 Concentration Represeting NET Formation

**Figure 2:**
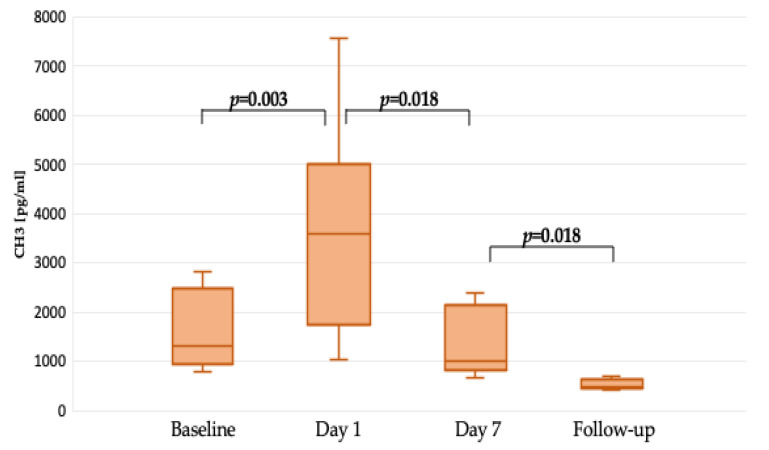
CH3 serum concentration differences between preoperative and postoperative values.

The median (Q1–3) follow-up time was 88 (49–143) days. Compared to preoperative results, on the first postoperative day, significantly higher CH3 concentrations following surgery were noticed (*p* = 0.003) as presented in Figure 2.

A significant difference between the first and seventh postoperative days’ CH3 serum concentration was noted, indicating decreased NET formation after LVAD implantation (*p* = 0.018), as presented in Figure 2.

The preoperative and control measurements performed within 88 (49–143) days following LVAD implantation presented a statistically significant CH3 decline (*p* < 0.001) as shown in Figure 2.

## 3. Discussion

Severe circulatory failure induces multiple organ failure through inflammation and hypoxia. The results of our study present changes in NET formation related to the severity of heart failure and response to the treatment. We observed increased plasma concentrations of citrullinated histone 3, describing NET formation in patients with advanced heart failure on optimal medical therapy. A strong correlation between preoperative CH3 and BNP concentrations was noticed. In the early postoperative period, a transient increase in CH3 was presented related to surgical intervention but followed by a subsequent decrease below the baseline concentration on the seventh postoperative day. The mechanical circulatory support of a left ventricular assist device decreases the risk for NETosis through circulatory improvement.

The innate immune system activation presented by neutrophil count secondary to heart failure was postulated in a CALIBER study [21]. The interactions between the implanted LVAD biomaterials and the host immune system were confirmed [22]. The aberrant states of monocyte and T-cell activation are claimed to be accompanied by two parallel processes in patients on mechanical support: selective loss of Th1 cytokine-producing CD4 T-cells through activation-induced cell death and unopposed activation of Th2 cytokine-producing CD4 T-cells resulting in B-cell hyperreactivity and dysregulated immunoglobulin synthesis [23]. The novelty of our research is based on decreased NETosis following LVAD implantation that indicates heart failure as a primary coagulatory and inflammatory condition. The presented results did not reveal a relation between mechanical support and innate immunological response contrary to progressive defects in cellular immunity, which were already reported [24,25].

Neutrophils are claimed to have a significant role in the development and progression of heart failure [26,27]. Neutrophils, platelets, and cytokines are involved in ischemic injury in myocardial infarction, stroke, and peripheral arterial disease [28]. Damaged heart tissue with abnormal cytokine release, reactive oxygen species, and hypoxia provides a further background for NET formation, either directly by activating neutrophils or via platelet activation. Platelets are sufficient promoters of NET formation on a thrombo-inflammatory basis. Several factors, including transforming growth factor (TGF) released from platelets and NETosis, stimulate cardiac fibrosis, which contributes to the progression of heart failure and exacerbates ventricular sclerosis and stiffness [29]. In the animal model by Zhao et al. [30], pressure overload induced non-ischemic cardiac failure and promoted neutrophil infiltration combined with NET formation. This phenomenon can lead to further heart damage including a decline in systolic and diastolic function [30]. In our analysis, the highest preoperative CH3 concentration was associated with a clinical diagnosis of heart failure and BNP concentration and decreased significantly to the lowest level after LVAD implantation in the follow-up. Langseth et al. [14] noticed significantly higher CH3 serum concentrations among patients with cardiogenic shock, which was related to heart failure and low cardiac output but not to infarct size. The NET formation burden in acute heart failure results from systemic hypoperfusion and organ hypoxia and reflects the intensity of inflammatory response in acute ischemia. Elevated levels of NET components in septic shock were speculated to be involved in cellular injury processes related to the causative pathogens [31,32]. Excessive NET formation may induce thrombosis and multiple organ failure. Increased risk of intravascular coagulation and multiorgan failure promotion was related to NET in critically ill patients [33]. Exaggerated NET formation may amplify inflammation and hinder healing [34].

Heart failure induces inflammatory and procoagulatory activation, which was measured by CH3 formation in our analysis. After mechanical support implantation, a significant drop in CH3 plasma concentration was noticed, which indicated circulatory improvement. As the CH3 derangements secondary to the surgical procedure were noticed on the first postoperative day, the significant CH3 decrease reflected improved hemodynamics secondary to the centrifugal pump. NETosis is induced by immune complexes, proinflammatory mediators, and activated endothelial cells [35]. NETs are characterized by highly proinflammatory properties with cytotoxic and prothrombotic effects [36]. Contrary to autoimmune diseases, tumors, or trauma [37], neutrophils in heart failure do not undergo apoptosis in NETosis, as mitochondrial DNA is released after reactive oxygen species activation [37].

Nucleic acids in NETs induce a coagulation cascade by factor XII and tissue factor [38]. The fibronectin and von Willebrand factor (VWF) bind into web-like structures after platelet activation by histones [39]. The role of NETs in LV remodeling following myocardial infarction and heart failure development processes [40] was investigated in contrast to their concentrations in chronic heart failure patients. Interestingly, in our analysis, the prothrombotic properties [41] of mechanical circulatory support combined with their proinflammatory effect [42] were not observed regarding NET formation. Despite nonphysiological blood circulation secondary to centrifugal pump implantation, the significance of circulatory improvement is superposed on an innate response to mechanical circulatory support. To the best of our knowledge, this is the first study explaining these issues of the primary role of heart failure in neutrophil activation.

As mechanical support is administered, the anticoagulation protocols are implemented to decrease the risk of thrombotic complications [43,44]. Following LVAD implantation, dual therapy based on oral vitamin K administration and antiplatelet drugs is recommended [45]. Mechanical circulatory support also induces coagulation cascade activation [46] combined with shear-mediated alteration of platelet function that provokes inflammatory activation. Satisfactory results of reduced anti-thrombotic therapy (RT) in patients with a left ventricular assist device (LVAD) were achieved [46] and individual risk was found to be related to genetic polymorphisms [47]. The previous reports suggested proinflammatory platelet–neutrophil interaction in LVAD patients [42]. In Ahmad et al.’s study [48], the decrease in inflammatory markers following LVAD implantation was related to an amino-terminal pro-B-type natriuretic peptide (NT-proBNP) drop, indicating heart failure-related inflammatory activation. We solely believe that the results of our analysis highlight that circulatory insufficiency is the primary NETosis factor. The LVAD-related circulatory improvement induced its significant reduction. More importantly, although LVAD as a mechanical device may induce inflammatory activation, circulatory improvement superposes the risk for NET formation.

Undoubtfully, the surgical intervention provoked CH3 escalation in the early postoperative period in the presented group. We found a significant CH3 upsurge in the presented group on the first postoperative day. It was in co-ordinance with a previous report [49], indicating an increase in circulating NETs during cardiac surgery, with postsurgical levels proportional to the cardiopulmonary bypass duration. Lesouhaitier et al. [49] indicated an increase in mature neutrophil count after surgery with cardiopulmonary bypass administration with enhanced NET release. We presented a reduction in progressive NET formation after LVAD implantation between the preoperative and early postoperative periods (first and seventh days) and in follow-up. Reducing NET formation may suggest a phase of inflammatory healing.

Several factors may have resulted in a decrease in NET formation after LVAD implantation in our study group. First, the abovementioned postoperative healing and improvement of cardiac output due to mechanical support are the most prominent causes. Importantly, all patients were treated with levosimendan infusion before LVAD implantation. The blood samples were obtained before drug initiation. Although levosimendan’s beneficial pleiotropic anti-inflammatory effects were described [50], previous studies [51] did not reveal a difference in circulating marker activation. Though we did not assess the direct influence of levosimendan on NET formation, we may suspect a positive effect of drug administration in the form of a decrease in CH3 concentration.

Moreover, after LVAD implantation, oral anticoagulation and antiplatelet (aspirin) therapy was implemented. The sufficient anticoagulation may also play a significant role in NET formation according to previous studies, as antithrombin III administration was proven beneficial [52]. The analysis of NET kinetics in acute coronary syndrome revealed a correlation with anti-thrombotic therapy [53]. In the animal model by Yoshimoto et al. [54], the dual antiplatelet therapy was related to NET formation decrease. In our analysis, the CH3 concentration progressively dropped during the follow-up. We suggest that besides the beneficial effect of mechanical circulatory support, anticoagulation and antiplatelet therapy may have a combined influence on the inflammatory milieu. Both levosimendan and anti-platelet and anti-thrombotic therapy should prevent thrombus formation or inflammatory effects which may accompany the artificial support, but may also act as confounding factors.

We conclude that changes in CH3 concentration reflect the degree of NET formation and thrombo-inflammatory characteristics of heart failure and healing related to medical and interventional therapy. Thus, CH3 may serve as a potential marker of progression or healing in heart failure patients. Further studies are necessary to improve our understanding of markers of NET in this specific group of patients and attempts to implement CH3 in contemporary diagnostics of heart failure.

### Study Limitation

This is a single-center prospective study performed on a limited number of patients undergoing LVAD procedures within 8 months. Due to the patients’ profile and the type of surgery, the limited number of enrolled participants is justified.

## 4. Materials and Methods

### 4.1. Patients and Method

There were 10 male patients with a median age of 61 (57–65) years enrolled in this prospective single-center analysis. They were referred for LVAD (Heartmate 3, Abbot Medical, Pleasanton, CA, USA) implantation due to advanced heart failure, estimated as INTERMACS 3. The demographical and clinical characteristics were collected and are presented in Table 3.

Blood samples for blood morphology and biochemical tests were collected at admission.

All patients were routinely followed up with clinical, echocardiographic and laboratory assessments.

### 4.2. NET Methodology

The concentration of CH3 was determined using the Human (CH3) Elisa Kit (Cat. No. 201-12-8862, Shanghai, China) from Shanghai Sunred Biological Technology Company Ltd. The solid phase (study wells) was precoated with human monoclonal anti-citrullinated histone C3 antibody. Then, simultaneous incubation of calibrators and patients’ samples with secondary anti-CH3 antibody labeled with biotin and combined with streptavidin-HRP was performed. An immune complex was than formed, and any unbound enzyme was removed during the washing step. Then, the substrate, tetramethylbenzidine (TMB), was added and incubated with the enzyme bound to the wells. The color first turned blue and then yellow as a result of adding the stopping solution. The intensity of the yellow color measured at a wavelength of 450 nm was directly proportional to the concentration of human citrullinated histone H3 in the samples. The concentration of CH3 in the samples was calculated using a regression line curve and the absorbance of specific CH3 calibrators.

### 4.3. Statistical Analysis

Continuous variables were reported as medians and interquartile ranges (Q1–Q3) since the data did not follow the normal distribution. Categorical data were presented as numbers and percentages. The comparison of interval parameters between both groups was performed by s Mann—Whitney test. Categorical data were compared by Fisher’s exact test. Statistical analysis was performed using JASP statistical software (JASP Team; 2023. Version 0.18.1 (https://jasp-stats.org, accessed on 20 August 2024)). *p* < 0.05 was considered statistically significant.

### 4.4. Bioethics Committee

The study was conducted in accordance with the Declaration of Helsinki and approved by the Institutional Ethics Committee of Poznan University of Medical Sciences, Poznan, Poland (protocol code 695/20 on 4 November 2020), for studies involving humans.

## 5. Conclusions

Neutrophil extracellular trap formation measured by citrullinated histone 3 concentration decreases after LVAD implantation. A perioperative increase is observed and may be explained by the surgical intervention and extracorporeal circulation administration.

## Figures and Tables

**Table 1:** Laboratory results.

Parameter	n = 10
Laboratory tests:	
1. Whole blood count analysis	
WBC (K/uL) (median (Q1–Q3))	7.23 (6.61–7.74)
Neutrophil (K/uL) (median (Q1–Q3))	4.80 (4.64–4.97)
Lymphocyte (K/uL) (median (Q1–Q3))	1.07 (0.92–1.71)
Monocyte (K/uL) (median (Q1–Q3))	0.55 (0.52–0.59)
LUC (K/uL) (median (Q1–Q3))	0.13 (0.08–0.13)
Hemoglobin (mmol/L) (median (Q1–Q3))	7.3 (7.1–9.0)
Hct (%) (median (Q1–Q3))	36 (34–43)
MCV (fl) (median (Q1–Q3))	88 (86–91)
MCHC (g/dL) (median (Q1–Q3))	20.79 (20.38–20.90)
NLR	4.5 (2.5–5.6)
MLR	0.36 (0.311–0.48)
SIRI	2.25 (1.27–2.61)
2. Liver function tests	
ALT (U/L) (median (Q1–Q3))	32 (20–45)
AST (U/L) (median (Q1–Q3))	26 (23–36)
3. Kidney function tests	
Creatinine (umol/L) (median (Q1–Q3))	146 (93–151)
Urea (mmol/L) (median (Q1–Q3))	8.9 (6.2–10.2)
Sodium (mmol/L) (median (Q1–Q3))	141 (138–142)
GFR (mL/min) (median (Q1–Q3))	48 (46–74)
4.. HF tests	
BNP (pg/mL) (median (Q1–Q3))	1321 (1024–2822)

Abbreviations: ALT—alanine aminotransferase; AST—aspartate aminotransferase; BNP—B-type natriuretic peptide; GFR—glomerular filtration rate, Hct—hematocrit; HF—heart failure; LUC—large unstained cell count; MCHC—mean corpuscular hemoglobin concentration; MCV—mean corpuscular volume; MLR—monocyte to lymphocyte ratio; n—number, NLR—neutrophil to lymphocyte ratio; SIRI—systemic inflammatory response index; Q—quartile, WBC—white blood cell count.

**Table 2:** Echocardiographic and RHC results.

Parameter	n = 10
Echocardiographic dimensions	
LV (mm) (median (Q1–Q3))	71 (60–75)
RV (mm) (median (Q1–Q3))	35 (33–38)
LA (mm) (median (Q1–Q3))	48 (45–57)
IVs (mm) (median (Q1–Q3))	9 (9–10)
LVEF (%) (median (Q1–Q3))	20 (17.5–26.0)
TAPSE (mm) (median (Q1–Q3))	15 (14–15)
RHC results:	
CI (l/min/m^2^) (median (Q1–Q3))	1.8 (1.7–2.0)
PVR (dynes/sec/cm^5^) (median (Q1–Q3))	260 (180–421)
PAP systolic (mmHg) (median (Q1–Q3))	41 (31–64)
PAP diastolic (mmHg) (median (Q1–Q3))	16 (13–29)
PAWP (mmHg) (median (Q1–Q3))	17 (14–26)
CO (L/min) (median (Q1–Q3))	3.62 (3.33–4.25)
SV (dynes/sec) (median (Q1–Q3))	54.8 (50–71.6)
SVR (dynes/sec/cm^5^) (median (Q1–Q3))	1661 (1372–1830)

Abbreviations: CI—cardiac index; cm—centimeter, CO—cardiac output; IVs—intraventricular septum; L—litre, LA—left atrium; LVEF—left ventricular ejection fraction; LV—left ventricular; mm—millimeter, mmHg—millimeters of mercury, n—number, PAP—pulmonary artery pressure; PAWP—pulmonary artery wedge pressure, PVR—pulmonary vascular resistance; sec—second; RHC—right heart catheterization; SV—stroke volume; SVR—systemic vascular resistance; TAPSE—tricuspid annular plane systolic excursion, Q—quartile.

**Table 3:** Patient characteristics.

Parameter	n = 10
Demographical:	
Age (years) (median (Q1–Q3))	61 (57–65)
Sex (M/F) (n/(%))	10/1
BMI (median (Q1–Q3))	28.4 (25.7–28.7)
Cardiomyopathy:	
ICM (n/(%))	6 (60)
DCM (n/(%))	4 (40)
Pulmonary hypertension (n/(%))	7 (70)
Co-morbidities:	
Arterial hypertension (n/(%))	2 (20)
AF persistent (n/(%))	5 (50)
AF paroxysmal (n/(%))	3 (30)
DM (n/(%))	5 (50)
Hypercholesterolenia (n/(%))	6 (60)
Kidney dysfunction * (n/(%))	5 (50)
Peripheral artery disease (n/(%))	2 (20)
Stroke (n/(%))	1 (10)

Abbreviations: AF—atrial fibrillation; BMI—body mass index; DCM—dilated cardiomyopathy; DM—diabetes mellitus; ICM—ischemic cardiomyopathy; Q—quartile. * glomerular filtration rate below 60 mL/min/1.73 m^2^.

## Data Availability

All data will be available for 3 years following the publication after reasonable request if presented in e-mail correspondence to the corresponding author.

## References

[1] Truby L.K., Rogers J.G. (2020). Advanced Heart Failure: Epidemiology, Diagnosis, and Therapeutic Approaches. JACC Heart Fail..

[2] Heidenreich P.A., Bozkurt B., Aguilar D., Allen L.A., Byun J.J., Colvin M.M., Deswal A., Drazner M.H., Dunlay S.M., Evers L.R. (2022). 2022 AHA/ACC/HFSA Guideline for the Management of Heart Failure: Executive Summary: A Report of the American College of Cardiology/American Heart Association Joint Committee on Clinical Practice Guidelines. Circulation.

[3] McDonagh T.A., Metra M., Adamo M., Gardner R.S., Baumbach A., Böhm M., Burri H., Butler J., Čelutkienė J., Chioncel O. (2022). 2021 ESC Guidelines for the diagnosis and treatment of acute and chronic heart failure: Developed by the Task Force for the diagnosis and treatment of acute and chronic heart failure of the European Society of Cardiology (ESC). With the special contribution of the Heart Failure Association (HFA) of the ESC. Eur. J. Heart Fail..

[4] Murphy S.P., Kakkar R., McCarthy C.P., Januzzi J.L. (2020). Inflammation in Heart Failure: JACC State-of-the-Art Review. J. Am. Coll. Cardiol..

[5] Siniarski A., Gąsecka A., Borovac J.A., Papakonstantinou P.E., Bongiovanni D., Ehrlinder H., Giustozzi M., Guerreiro R.A., Parker W.A.E. (2023). Blood Coagulation Disorders in Heart Failure: From Basic Science to Clinical Perspectives. J. Card. Fail..

[6] Castanheira F.V.S., Kubes P. (2019). Neutrophils and NETs in modulating acute and chronic inflammation. Blood.

[7] Tang X., Wang P., Zhang R., Watanabe I., Chang E., Vinayachandran V., Nayak L., Lapping S., Liao S., Madera A. (2022). KLF2 regulates neutrophil activation and thrombosis in cardiac hypertrophy and heart failure progression. J. Clin. Investig..

[8] Zhang Y., Deng X., Zhang J., Zhang L., Akram Z., Zhang B., Sun S. (2022). A Potential Driver of Disseminated Intravascular Coagulation in Heat Stroke Mice: Neutrophil Extracellular Traps. Int. J. Environ. Res. Public Health.

[9] Tsigkou V., Oikonomou E., Anastasiou A., Lampsas S., Zakynthinos G.E., Kalogeras K., Katsioupa M., Kapsali M., Kourampi I., Pesiridis T. (2023). Molecular Mechanisms and Therapeutic Implications of Endothelial Dysfunction in Patients with Heart Failure. Int. J. Mol. Sci..

[10] Chung I., Choudhury A., Patel J., Lip G.Y. (2009). Soluble, platelet-bound, and total P-selectin as indices of platelet activation in congestive heart failure. Ann. Med..

[11] Halade G.V., Lee D.H. (2022). Inflammation and resolution signaling in cardiac repair and heart failure. EBioMedicine.

[12] Bai B., Xu Y., Chen H. (2023). Pathogenic roles of neutrophil-derived alarmins (S100A8/A9) in heart failure: From molecular mechanisms to therapeutic insights. Br. J. Pharmacol..

[13] Brinkmann V., Reichard U., Goosmann C., Fauler B., Uhlemann Y., Weiss D.S., Weinrauch Y., Zychlinsky A. (2004). Neutrophil extracellular traps kill bacteria. Science.

[14] Langseth M.S., Andersen G.Ø., Husebye T., Arnesen H., Zucknick M., Solheim S., Eritsland J., Seljeflot I., Opstad T.B., Helseth R. (2020). Neutrophil extracellular trap components and myocardial recovery in post-ischemic acute heart failure. PLoS ONE.

[15] Moschonas I.C., Tselepis A.D. (2019). The pathway of neutrophil extracellular traps towards atherosclerosis and thrombosis. Atherosclerosis.

[16] Yipp B.G., Petri B., Salina D., Jenne C.N., Scott B.N., Zbytnuik L.D., Pittman K., Asaduzzaman M., Wu K., Meijndert H.C. (2012). Infection-induced NETosis is a dynamic process involving neutrophil multitasking in vivo. Nat. Med..

[17] Obama T., Itabe H. (2020). Neutrophils as a Novel Target of Modified Low-Density Lipoproteins and an Accelerator of Cardiovascular Diseases. Int. J. Mol. Sci..

[18] Masuda S., Nakazawa D., Shida H., Miyoshi A., Kusunoki Y., Tomaru U., Ishizu A. (2016). NETosis markers: Quest for specific, objective, and quantitative markers. Clin. Chim. Acta.

[19] Li P., Li M., Lindberg M.R., Kennett M.J., Xiong N., Wang Y. (2010). PAD4 is essential for antibacterial innate immunity mediated by neutrophil extracellular traps. J. Exp. Med..

[20] Sørensen O.E., Borregaard N. (2016). Neutrophil extracellular traps—The dark side of neutrophils. J. Clin. Investig..

[21] Shah A.D., Denaxas S., Nicholas O., Hongorani A.D., Hemingway H. (2017). Neutrophil counts and initial presentation of 12 cardiovascular diseases: A CALIBER cohort study. J. Am. Coll. Cardiol..

[22] Itescu S., Schuster M., Burke E., Ankersmit J., Kocher A., Deng M., John R., Lietz K. (2003). Immunobiologic consequences of assist devices. Cardiol. Clin..

[23] Itescu S., John R. (2003). Interactions between the recipient immune system and the left ventricular assist device surface: Immunological and clinical implications. Ann. Thorac. Surg..

[24] Schuster M., Kocher A., John R., Hoffman M., Ankersmit J., Lietz K., Edwards N., Oz M., Itescu S. (2002). B-cell activation and allosensitization after left ventricular assist device implantation is due to T-cell activation and CD40 ligand expression. Hum. Immunol..

[25] Ankersmit H.J., Tugulea S., Spanier T., Weinberg A.D., Artrip J.H., Burke E.M., Flannery M., Mancini D., Rose E.A., Edwards N.M. (1999). Activation-induced T-cell death and immune dysfunction after implantation of left-ventricular assist device. Lancet.

[26] Li X., Xu C., Li Q., Shen Q., Zeng L. (2023). Exploring key genes associated with neutrophil function and neutrophil extracellular traps in heart failure: A comprehensive analysis of single-cell and bulk sequencing data. Front. Cell Dev. Biol..

[27] Urbanowicz T.K., Olasińska-Wiśniewska A., Michalak M., Straburzyńska-Migaj E., Jemielity M. (2020). Neutrophil to lymphocyte ratio as noninvasive predictor of pulmonary vascular resistance increase in congestive heart failure patients: Single-center preliminary report. Adv. Clin. Exp. Med..

[28] Sorvillo N., Cherpokova D., Martinod K., Wagner D.D. (2019). Extracellular DNA NET-Works With Dire Consequences for Health. Circ. Res..

[29] Saadat S., Noureddini M., Mahjoubin-Tehran M., Nazemi S., Shojaie L., Aschner M., Maleki B., Abbasi-Kolli M., Rajabi Moghadam H., Alani B. (2021). Pivotal Role of TGF-β/Smad Signaling in Cardiac Fibrosis: Non-coding RNAs as Effectual Players. Front. Cardiovasc. Med..

[30] Martinod K., Witsch T., Erpenbeck L., Savchenko A., Hayashi H., Cherpokova D., Gallant M., Mauler M., Cifuni S.M., Wagner D.D. (2017). Peptidylarginine deiminase 4 promotes age-related organ fibrosis. J. Exp. Med..

[31] Maruchi Y., Tsuda M., Mori H., Takenaka N., Gocho T., Huq M.A., Takeyama N. (2018). Plasma myeloperoxidase-conjugated DNA level predicts outcomes and organ dysfunction in patients with septic shock. Crit. Care.

[32] Li R.H.L., Tablin F. (2018). A Comparative Review of Neutrophil Extracellular Traps in Sepsis. Front. Vet. Sci..

[33] Abrams S.T., Morton B., Alhamdi Y., Alsabani M., Lane S., Welters I.D., Wang G., Toh C.H. (2019). A Novel Assay for Neutrophil Extracellular Trap Formation Independently Predicts Disseminated Intravascular Coagulation and Mortality in Critically Ill Patients. Am. J. Respir. Crit. Care Med..

[34] Zhu S., Yu Y., Ren Y., Xu L., Wang H., Ling X., Jin L., Hu Y., Zhang H., Miao C. (2021). The emerging roles of neutrophil extracellular traps in wound healing. Cell Death Dis..

[35] Vulesevic B., Sirois M.G., Allen B.G., de Denus S., White M. (2018). Subclinical Inflammation in Heart Failure: A Neutrophil Perspective. Can. J. Cardiol..

[36] Mayadas T.N., Cullere X., Lowell C.A. (2014). The multifaceted functions of neutrophils. Annu. Rev. Pathol..

[37] Tao G., Liao W., Hou J., Jiang X., Deng X., Chen G., Ding C. (2024). Advances in crosstalk among innate immune pathways activated by mitochondrial DNA. Heliyon.

[38] Fuchs T.A., Brill A., Duerschmied D., Schatzberg D., Monestier M., Myers D.D., Wrobleski S.K., Wakefield T.W., Hartwig J.H., Wagner D.D. (2010). Extracellular DNA traps promote thrombosis. Proc. Natl. Acad. Sci. USA.

[39] von Brühl M.L., Stark K., Steinhart A., Chandraratne S., Konrad I., Lorenz M., Khandoga A., Tirniceriu A., Coletti R., Köllnberger M. (2012). Monocytes, neutrophils, and platelets cooperate to initiate and propagate venous thrombosis in mice in vivo. J. Exp. Med..

[40] Wu Y., Wei S., Wu X., Li Y., Han X. (2023). Neutrophil extracellular traps in acute coronary syndrome. J. Inflamm..

[41] Liesdek O.C.D., Urbanus R.T., de Maat S., de Heer L.M., Ramjankhan F.Z., Sebastian S.A.E., Huisman A., de Jonge N., Vink A., Fischer K. (2023). Insights in the Prothrombotic Changes after Implantation of a Left Ventricular Assist Device in Patients with End-Stage Heart Failure: A Longitudinal Observational Study. ASAIO J..

[42] Granja T., Magunia H., Schüssel P., Fischer C., Prüfer T., Schibilsky D., Serna-Higuita L., Wendel H.P., Schlensak C., Häberle H. (2022). Left ventricular assist device implantation causes platelet dysfunction and proinflammatory platelet-neutrophil interaction. Platelets.

[43] Jorde U.P., Saeed O., Koehl D., Morris A.A., Wood K.L., Meyer D.M., Cantor R., Jacobs J.P., Kirklin J.K., Pagani F.D. (2024). The Society of Thoracic Surgeons Intermacs 2023 Annual Report: Focus on Magnetically Levitated Devices. Ann. Thorac. Surg..

[44] Tang S., Xu L., Li H., Wu Z., Wen Q. (2023). Anticoagulants in adult extracorporeal membrane oxygenation: Alternatives to standardized anticoagulation with unfractionated heparin. Eur. J. Clin. Pharmacol..

[45] Trachtenberg B., Cowger J., Jennings D.L., Grafton G., Loyaga-Rendon R., Cogswell R., Klein L., Shah P., Kiernan M., Vorovich E. (2023). HFSA Expert Consensus Statement on the Medical Management of Patients on Durable Mechanical Circulatory Support. J. Card. Fail..

[46] Marshall D., Sanchez J., Yuzefpolskaya M., Sayer G.T., Takeda K., Naka Y., Colombo P.C., Uriel N., Topkara V.K. (2021). Safety of reduced anti-thrombotic strategy in patients with HeartMate 3 left ventricular assist device. J. Heart Lung Transplant..

[47] Zhalbinova M.R., Rakhimova S.E., Kozhamkulov U.A., Akilzhanova G.A., Chinybayeva A.A., Akilzhanov K.R., Shaimardanov N.K., Kuanysheva A.G., Lee J.H., Kairov U.Y. (2023). Role of Genetic Polymorphisms in the Development of Complications in Patients with Implanted Left Ventricular Assist Devices: HeartWare, HeartMate II, and HeartMate 3. J. Clin. Med..

[48] Ahmad T., Wang T., O’Brien E.C., Samsky M.D., Pura J.A., Lokhnygina Y., Rogers J.G., Hernandez A.F., Craig D., Bowles D.E. (2015). Effects of left ventricular assist device support on biomarkers of cardiovascular stress, fibrosis, fluid homeostasis, inflammation, and renal injury. JACC Heart Fail..

[49] Lesouhaitier M., Gregoire M., Gacouin A., Coirier V., Frerou A., Piau C., Cattoir V., Dumontet E., Revest M., Tattevin P. (2022). Neutrophil function and bactericidal activity against Staphylococcus aureus after cardiac surgery with cardiopulmonary bypass. J. Leukoc. Biol..

[50] Cheko J., Patsalis N., Kreutz J., Divchev D., Chatzis G., Schieffer B., Markus B. (2023). The Impact of Positive Inotropic Therapy on Hemodynamics and Organ Function in Acute Heart Failure: A Differentiated View. J. Pers. Med..

[51] Husebye T., Eritsland J., Arnesen H., Bjørnerheim R., Mangschau A., Seljeflot I., Andersen G.Ø. (2014). Association of interleukin 8 and myocardial recovery in patients with ST-elevation myocardial infarction complicated by acute heart failure. PLoS ONE.

[52] Ishikawa M., Yamashita H., Oka N., Ueda T., Kohama K., Nakao A., Kotani J. (2017). Antithrombin III improved neutrophil extracellular traps in lung after the onset of endotoxemia. J. Surg. Res..

[53] Ferré-Vallverdú M., Latorre A.M., Fuset M.P., Sánchez E., Madrid I., Ten F., Vallés J., Santos M.T., Bonanad S., Moscardó A. (2022). Neutrophil extracellular traps (NETs) in patients with STEMI. Association with percutaneous coronary intervention and antithrombotic treatments. Thromb. Res..

[54] Yoshimoto M., Kagawa S., Kajioka H., Taniguchi A., Kuroda S., Kikuchi S., Kakiuchi Y., Yagi T., Nogi S., Teraishi F. (2023). Dual antiplatelet therapy inhibits neutrophil extracellular traps to reduce liver micrometastases of intrahepatic cholangiocarcinoma. Cancer Lett..

